# Comparison of Electrical Impedance Tomography and Ultrasonography for Determination of Solid and Cystic Lesion Resembling Breast Tumor Embedded in Chicken Phantom

**DOI:** 10.2478/joeb-2021-0008

**Published:** 2021-11-20

**Authors:** L. Choridah, D. Kurniadi, K. Ain, M.F. Ulum, U. Mukhaiyar, A.D. Garnadi, N.H. Setyawan

**Affiliations:** 1 Faculty of Medicine, Public Health and Nursing, Universitas Gadjah Mada, Yogyakarta, Indonesia; 2 Physics Engineering, Institut Teknologi Bandung, Bandung, Indonesia; 3 Physics, Universitas Airlangga, Surabaya, Indonesia; 4 Animal Medicine,Institut Pertanian Bogor, Bogor, Indonesia; 5 Statistics, Institut Teknologi Bandung, Bandung, Indonesia; 6 Mathematics and Sciences, Institut Pertanian Bogor, Bogor, Indonesia

**Keywords:** Electrical impedance, ultrasound, phantom

## Abstract

Ultrasonography (US) and Electrical Impedance Tomography (EIT) can be used to detect breast cancer. Ultrasonography is based on non-ionizing radiations without adverse biological effects. A set of electrodes was placed around the torso and a small alternating current (AC) was injected via two of the electrodes into the object. This study aimed to acquire preliminary data to evaluate the EIT method for differentiation of artificial solid and cystic tumors in comparison to standard US.

This study used a phantom made from chicken meat. In order to obtain the image of the solid tumor, an olive with carrot insertion was done, and the cystic tumor was created by filling a small balloon with water. GE Logic C5 ultrasound was performed with a 12 MHz linear transducer. For EIT measurement, 16 ECG electrodes and 32 ECG electrodes were placed. Data processing was done using the Graz consensus Reconstruction algorithm for EIT (GREIT) and Newton's One Step Error Reconstructor (NOSER) methods.

The artificial solid tumor produced an ultrasound image of an oval, inhomogeneous lesions. The GREIT method with 16 electrodes of artificial solid tumor did not show a match between the reconstructed image and the original object containing 2 anomalies, but a match was found with 32 electrodes. In the NOSER method, both 16 and 32 electrodes showed a match. Ultrasound of the artificial cystic tumor showed an oval, circumscribed, anechoic with posterior enhancement. Both the GREIT and NOSER methods using the artificial cystic tumor showed a match between the reconstructed image and the original object containing two anomalies.

EIT has a lower imaging resolution in comparison to ultrasonography, but is progressively maturing as a tool for monitoring and imaging. The solid and cystic anomalies on the phantoms were visualized by the GREIT and NOSER methods except for the solid anomaly with the GREIT 16 electrodes.

## Introduction

Worldwide, breast malignancy is the leading invasive cancer of women population. It is estimated that breast cancer comprises 24.5% and 15.5% of new cancer cases and number of cancer deaths in female in 2020, respectively [1,2]. Approximately 627,000 deaths worldwide were attributable to breast cancer in 2018 [1]. Therefore, early detection of breast malignancy is very important. Medical imaging devices such as the well-known Ultrasonography (US) and Electrical Impedance Tomography (EIT) can be utilized to detect breast cancer. This study presents new findings related to phantom applications and discusses the comparison of US and EIT for identification of solid and cystic lesions resembling breast tumors embedded in an experimental chicken phantom.

US is a medical modality using the principles of high-frequency acoustic waves to create a projection of a human’s internal structure. US is routinely used to detect several breast changes including lumps and density. Abnormality detected in mammogram can also be confirmed with ultrasound [3]. Ultrasound has particular advantages in differentiating between fluid-filled cysts, which are usually benign and solid lesions that may need further investigation to diagnose cancer. Another advantage of ultrasound is the applicability in guiding biopsy into a certain suspected area for cytology or histology examination for the presence of cancer cells. This sonography-guided biopsy can also be performed in the axillary lymph nodes [3].

Ultrasonography, based on non-ionizing radiation, is a dynamic imaging technique with the ability for real-time visualization. The image of body structures is produced by recording the echoes of ultrasonic wave pulses that are reflected back by tissue planes without alteration of the tissue density. The process largely depends on the tissue acoustic impedance. The ultrasound waves are released by an electrically stimulated piezo-electric crystal called a transducer. The images displayed on computer systems are electrical impulse conversions of sound waves that are bounced back to the transducer in the form of echoes by tissue boundaries with various acoustic impedance. The interpretation of the results then categorizes the data into anechoic, hypoechoic, and hyperechoic. Principally, ultrasound works by the fact of differences of acoustic impedance among various tissues [4].

Ultrasonography, which does not expose a person to ionizing radiation, is widely available, easy to obtain, and relatively affordable compared to other imaging modalities. However, ultrasound examination is operator-dependent, hence the operator has to complete a certain degree of training to be able to produce reliable and standard-conforming results [5].

EIT, as an imaging modality, allows us to estimate the distribution of electrical parameters such as resistivity from electrical measurements on the periphery of the object. EIT is very safe as an imaging modality and also affordable, because it requires relatively simple hardware and does not use ionizing radiation. In EIT, an alternating current is injected into the object, and the induced voltages are measured. The measurement data are introduced to a computer for reconstructing the image of the resistivity distribution. EIT is a promising technique for medical applications such as breast cancer detection.

EIT is being studied as an imaging technique to differentiate solid and liquid structures. Several studies have investigated the use of EIT in the setting of intensive care to evaluate lung function. In these studies, the authors mapped the electrodes around the patients’ body surface and injected alternating current (AC) in small amounts through two of the electrodes. The remaining electrodes pair then can be utilized to measure the generated surface voltage. These data were then used to map the distribution of impedance in the cross-sectional plane in the body region, especially for the lungs and thoracic tissues.

There are no definitive studies that show a significant difference between lung organ tissue and solid and cystic chicken tumor phantoms with respect to electrical resistivity. In addition to the anatomical and functional differences, lung tissue is normally filled with air, while the chicken phantom is filled with an artificial solid tumor made from an olive with carrot and an artificial cystic tumor from a water balloon. From the material viewpoint, air is an excellent isolator, while olive and water balloon have similar electrical conductivity as their surrounding structures. This conductivity difference may lead to problems in imaging artificial solid and cystic tumors by the EIT method. The best imaging accuracy is achieved when there is substantial impedance differences between the medium/lesion under investigation and surrounding structures, especially for solid and cystic tumors. Nevertheless, our first attempt revealed promising outcomes and the impedance mapping showed linear correlations with the ultrasound image.

From previous studies, EIT has been used extensively with relatively known electrodes placement and signal analysis to evaluate lung structures. However, there is no data available regarding optimal settings for EIT to be able to differentiate between solid and cystic tumors. This preliminary study will seek to acquire new data to assess EIT performance to differentiate solid and cystic lesions with comparison of ultrasound images.

## Materials and methods

**Fig.1:**
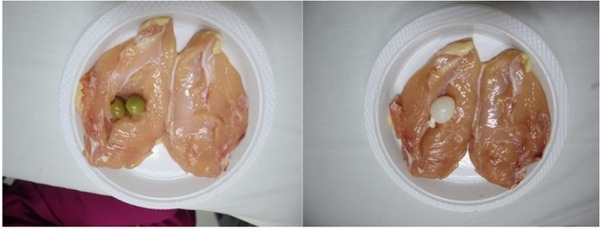
Chicken meat phantom with olive filled with carrot as artificial solid tumor (two green olives) and small balloon filled with water as artificial cystic tumor.

This prospective investigational study used a phantom made of chicken meat. A phantom from chicken meat with a depth of 4 cm was used to create ultrasound images that resemble breast tissue. In order to obtain the image of the solid tumor, an olive insertion filled with carrot was used. An artificial cystic tumor was created by filling a chicken phantom with a small balloon filled with water (Fig. 1).

**Fig.2:**
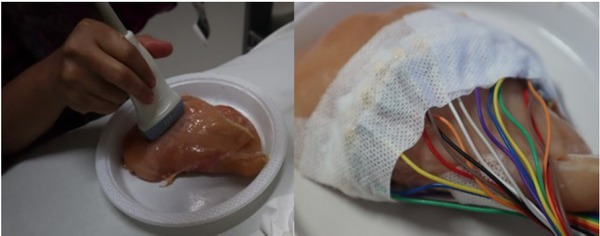
Ultrasound of chicken meat phantom using linear transducer and EIT with electrodes.

Ultrasound scans of artificial solid and cystic tumor were performed using a GE Logic C5 ultrasound machine with a 12 MHz linear transducer. For EIT measurements, 16 ECG electrodes and 32 ECG electrodes were placed on the chicken phantom (Fig.2). Data processing was done using the Graz consensus Reconstruction algorithm for EIT (GREIT) and Newton's One Step Error Reconstructor (NOSER) methods.

### Ethical approval

The conducted research is not related to either human or live animal use, therefore no ethical approval was needed.

## Results

**Fig.3:**
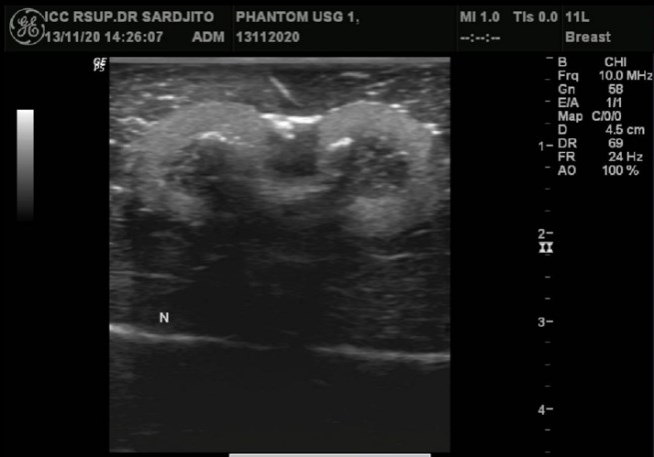
Ultrasound images of two artificial solid tumors made of olive and carrot pieces to represent oval inhomogeneous lesions. Olive flesh appeared as a mildly hyperechoic area at the edge of the lesion, while carrot pieces appeared as a hypoechoic area in the middle portion with a posterior shadow and several small linear hyperechoic areas.

An artificial solid tumor made of olive filled with carrot slices give an ultrasound image of oval, inhomogeneous lesions. Olive flesh appears as a mild hyperechoic area at the edge of the lesion and carrot pieces appear in the middle as a hypoechoic lesion with a posterior shadow and small linear hyperechoic area (Fig. 3).

**Fig.4:**
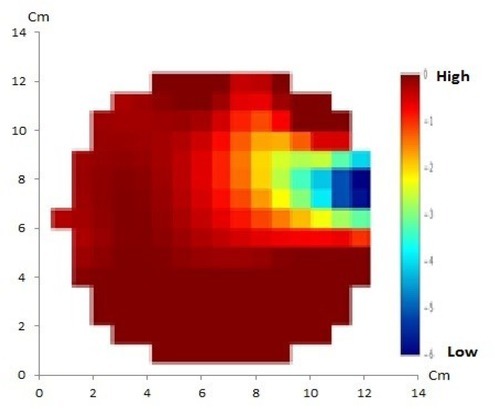
GREIT method with 16 electrodes of artificial solid tumor shows discordance between the reconstructed image and the original object in which only one anomaly is displayed instead of two.

**Fig.5:**
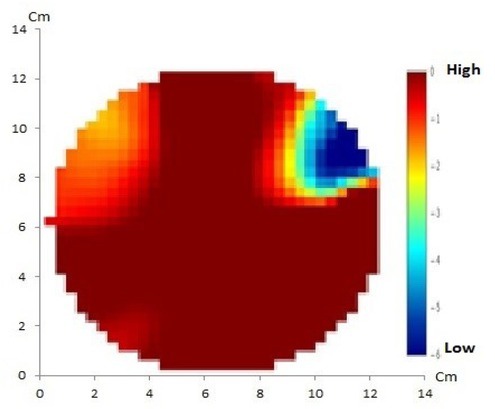
GREIT method with 32 electrodes of artificial solid tumor shows a match between the reconstructed image and the original object containing two anomalies.

EIT using the GREIT method with 16 and 32 electrodes of artificial solid tumor are shown in Fig. 4 and Fig. 5, respectively.

**Fig.6:**
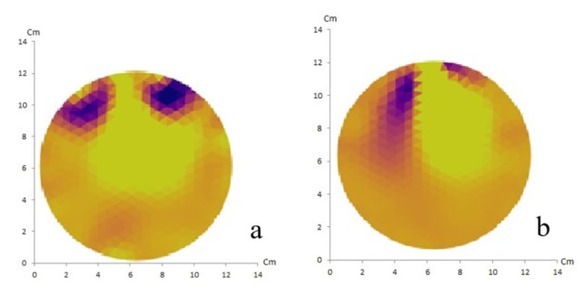
NOSER method with 16 electrodes (a) and 32 electrodes (b) of artificial solid tumor ion shows a match between the reconstructed image and the original object containing two anomalies.

EIT using the NOSER method with 16 and 32 electrodes of artificial solid tumor is shown in Fig. 6.

**Fig.7:**
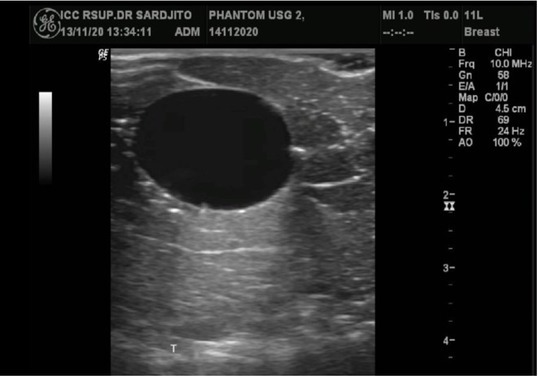
A water-filled balloon was used to mimic a cystic lesion. This ultrasound image demonstrated the cystic lesion as a well-defined, oval-shaped anechoic area with posterior enhancement.

Ultrasound imagery of the artificial cystic tumor originating from the small water-filled balloon (Fig. 7) showed an oval, well-circumscribed, anechoic appearance with posterior enhancement.

**Fig.8:**
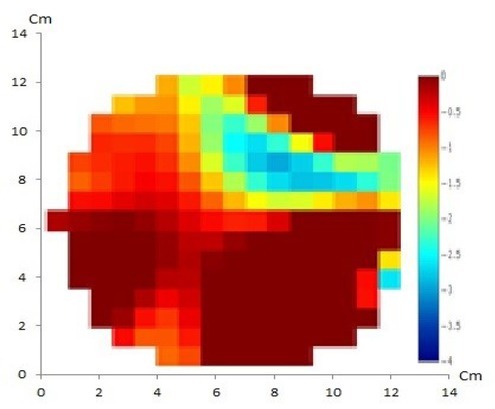
GREIT method with 16 electrodes of artificial cystic tumor has shown a match between the reconstructed image and the original object containing one anomaly.

**Fig.9:**
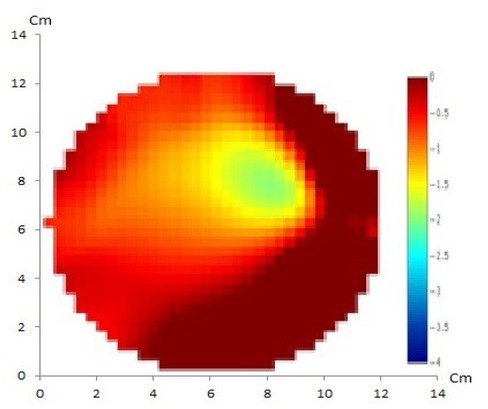
GREIT method with 32 electrodes of artificial cystic tumor showed a match between the reconstructed image and the original object containing one anomaly that shows clear oval with smooth borders resembling the real cystic tumor.

**Fig.10:**
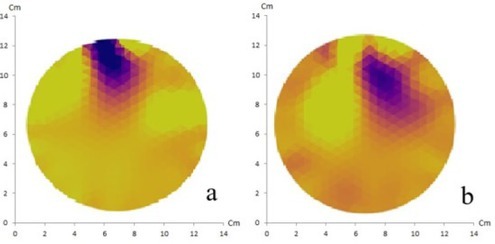
NOSER method with 16 (a) and 32 electrodes (b) of artificial cystic tumor shows a match between the reconstructed image and the original object containing one anomaly.

The artificial cystic tumor was visualized using the GREIT method with 16 and 32 electrodes, shown in Fig. 8 and Fig. 9, respectively EIT images using the NOSER method with 16 and 32 electrodes of artificial cystic tumor are also shown in Fig. 10.

## Discussion

Each tissue type of a living organism has different sound reflectivity due to underlying differences in their micro-structures, which is known as echogenicity. These differences in echogenicity make the famous grayscale images in ultrasound examination and can be categorized as hyperechoic (which appears white/bright on the screen), hypoechoic (which appears darker or gray on the screen), and anechoic (which appears black/dark on the screen) [5].

### Comparison of EIT and ultrasonography images of the artificial solid tumor

The artificial solid tumor made of olive filled with carrot slices gave an ultrasound image of an oval, inhomogeneous lesions. Olive flesh appeared as a mildly hyperechoic area at the edge of the lesion and carrot pieces appeared in the middle as a hypoechoic area. Acoustic shadow from the central area of this artificial solid tumor was caused by increased attenuation in the object compared to surrounding structures. The very white area on top of the lesion was most likely due to specular reflection. The periphery of the lesion that appeared as a hyperechoic area originating from olive flesh has an echostructure resembling that of a solid breast tumor, namely a lipoma.

Lipoma is a benign tumor which has characteristics of slowly growing neoplasm with well-defined margins. Most patients’ complaints involve asymmetrical focal increase of involved breast region without any mass being able to be palpated. Upon compression using a US transducer, lipoma tends to be a soft tumor and easily deformed. US images usually reveal the appearance of lipoma as a thin capsule mass with variable echogenic structure, accompanied by lamellar or stippled features [6]. The central part is a hypoechoic lesion with an acoustic shadow similar to that of a malignant tumor. Notable features of malignant tumor in US are hypoechoic or very hypoechoic lesion; taller-than-wide shape; irregular or spiculated margin; micro-calcifications; and posterior acoustic shadowing [6].

In this EIT experiment, 16 and 32 electrodes were used with the GREIT and NOSER reconstruction methods. It appears that the NOSER method can produce reconstructed images with higher resolution when compared to the GREIT method. From the four reconstruction results, it appears that there were two objects corresponding to the number of anomalies in the phantom, however the GREIT method with 16 electrodes did not show a match between the reconstructed image and the original object containing two anomalies.

### Comparison of EIT and ultrasonography images of artificial cystic tumor

Ultrasonography of an artificial cystic tumor originating from a small water-filled balloon showed an oval, circumscribe, anechoic appearance with posterior enhancement. The posterior enhancement is due to the smaller attenuation in water/cystic object compared to surrounding structures. Phantom cystic tumors made of water-filled balloons have the appearance of a breast cystic tumor.

Cystic lesions are the most common cause for complaints of lumps in the breast in women less than 50 years. The accumulation of fluid occurs due to blockage of terminal ducts in extralobular region due to various causes (e.g. fibrosis or epithelial proliferation in the duct). During US examination, breast cysts appear as solitary or multiple anechoic regions with round-to-oval shape, well-defined margin, thin-walled, and posterior acoustic enhancement [6].

In this EIT experiment, 16 and 32 electrodes were used with the GREIT and NOSER reconstruction method. GREIT is primarily used as reconstruction algorithm for lung imaging using EIT. There are three bases for this algorithm, which are: 1). “ingredients” and evaluation method selection, 2) GREIT variants experience and evaluation, and 3) GREIT algorithm consensus and definition. The criteria for algorithms evaluation are: a) quantitative output for all positions, b) low and uniform of reconstructed position error, c) good resolution in terms of small PSF, uniform, and few artefacts, d) excellent noise performance, e) low sensitivity to boundary and electrode motion, and f) excellent performance on experimental and clinical data. This consensus represents a major group of experts in EIT algorithms and its clinical applications [7].

To reconstruct the EIT data for imaging purpose, mapping is needed between electrical conductivity in the interior of human body and the voltage distribution in the body surface. This mapping used a finite-element model and Gauss-Newton method using NOSER to solve the inverse problem. Minimal processing time requirement and accuracy requirement should be considered when using the NOSER calculation [8]. In this study, the NOSER method produced reconstructed images with higher resolution when compared to the GREIT method. From the four reconstruction results, it appeared that there was one object corresponding to the number of anomalies in the phantom.

The strength of ultrasound imaging is its ability to delineate areas of sharp acoustic density changes. However, there are circumstances where ultrasound reflection signal cannot be used confidently to determine the boundaries, such as masking from surrounding area or being outside of the ultrasound beam. On the other hand, EIT inherently contains only low-quality information, but is able to convey complete data of region covered by the electrodes [9]. It is clear that in terms of diagnostics, ultrasound has higher imaging resolution in comparison with EIT. Recently, EIT is increasingly studied and utilized as a imaging and monitoring tool.

The study by Leonhauser *et al*. showed that for estimation of residual urine (RU), EIT-cystovolumetry performs well in comparison to standard non-invasive ultrasound. Clearly, for this volumetric estimation, the placement configuration of the electrodes plays a significant role. Motion artifacts of the abdomen should also be considered when developing the algorithm [10].

The limitation of this study is mainly due to the limited electrodes configuration and electrical current variation. Chicken phantom tissue structures may also not represent real living human tissue, and true solid or cystic tumoral lesion of human may also behave differently than our artificial lesions. The strength of this study is its ability to gather preliminary data of EIT as an imaging tool to differentiate between solid and cystic lesions, when compared to standard ultrasound technique. Clinical implication of EIT imaging can be very beneficial, such as for reducing the operator-dependency of certain imaging modality, increasing structures or lesion delineation, or even integrating physiological properties of a lesion to the anatomical data gathered from standard structural imaging tools such as ultrasound. Future investigation regarding protocol optimization and correlational study is needed to bring EIT to clinical use.
